# Synthesis of Nanocrystalline Mg-Al Hydrotalcites in the Presence of Starch—the Effect on Structure and Composition

**DOI:** 10.3390/ma13030602

**Published:** 2020-01-29

**Authors:** Alicja Michalik, Bogna D. Napruszewska, Anna Walczyk, Joanna Kryściak-Czerwenka, Dorota Duraczyńska, Ewa M. Serwicka

**Affiliations:** Jerzy Haber Institute of Catalysis and Surface Chemistry, Niezapominajek 8, 30-239 Krakow, Poland; ncmichal@cyf-kr.edu.pl (A.M.); ncnaprus@cyf-kr.edu.pl (B.D.N.); ncawalczyk@cyf-kr.edu.pl (A.W.); ncduracz@cyf-kr.edu.pl (D.D.)

**Keywords:** hydrotalcite, layered double hydroxide, biotemplate, starch, nanoparticles, crystallinity

## Abstract

The study describes the synthesis of Mg-Al hydrotalcite (Ht) with the use of starch as a structure controlling biotemplate. Syntheses were carried out at room temperature, by co-precipitation at pH = 10. The investigated synthesis parameters included the nature of the precipitating agent (NaOH/Na_2_CO_3_ or NH_3_aq/(NH_4_)_2_CO_3_), the nature of starch (potato, corn and cassava), the method of starch addition to reagents, the method of drying and the effect of washing. The materials were examined with X-ray diffraction, scanning electron microscopy/energy dispersive X-ray spectroscopy and infrared spectroscopy. The data show that synthesis of Ht materials in the presence of starch, with use of the ammonia-based precipitant, enabled preparation of nanocrystalline Ht with very fine (<50 nm) particle size. All investigated starches had a similar effect on the crystallinity and the grain size of Ht precipitates. Ht with the smallest nanocrystals was obtained when starch was present in all solutions used for synthesis, and the final product subjected to freeze drying. Washing with water was found to enhance recrystallization and exchange of nitrates for carbonates. Infrared spectra showed that an interaction exists between the biopolymer template and the Ht particles, resulting in a higher degree of order within the Ht-adhering starch component.

## 1. Introduction

Synthetic hydrotalcite-like materials (Ht), related to the naturally occurring mineral of chemical composition [(Mg_0.75_Al_0.25_)(OH)_2_](CO_3_)_0.125_·0.5H_2_O, are also referred to as layered double hydroxides (LDHs), or anionic clays [1]. The layered Ht structure is extremely flexible and may accommodate a number of divalent M^2+^ and trivalent M^3+^ cations other than Mg^2+^ and Al^3+^, in a variable atomic ratio, as well as anions other than carbonate. In consequence, the Ht materials are described by the general formula [M^2+^_1−x_M^3+^_x_(OH)_2_]^x+^(A^n−^_x/n_)·mH_2_O, where M^2+^ and M^3+^ are the elements forming the hydroxide layer, A^n-^ is the interlayer anion compensating the layer charge, and H_2_O refers to the interlayer water. Ht materials find numerous applications as catalysts, adsorbents, medicines and medicine carriers, polymer fillers, etc. [1,2,3,4,5,6,7,8,9]. Of particular importance are nanostructural forms of Ht with controllable particle size, morphology, crystallinity and texture. However, although Ht have the advantage of being easily prepared in laboratory, the conventional methods of their synthesis, e.g., co-precipitation at a constant pH, yield generally precipitates composed of aggregated particles, and offer only limited control over textural properties. Among advanced synthetic procedures developed with aim to prepare Ht materials with carefully designed nanostructure, the most promising ones are those based on the concept of structure templating [7]. The advantages of templating strategy are good reproducibility, possibility of large-scale synthesis and abundance of viable structure-directing templates.

Due to the increasing awareness towards biocompatibility of synthetic procedures, a new trend in template mediated synthesis of Ht materials emerged in the last few years, based on the use of naturally occurring biomaterials for engineering of novel nanostructures [7]. The biotemplating approach takes advantage of intricate morphology, hierarchical inner structure and surface functionalities of natural materials. The method is considered particularly attractive owing to the low cost, renewable nature, biodegradable character, and environmental compatibility of natural reagents. A literature search shows that synthesis of Ht with the use of sacrificial biotemplates is a very recent tendency, with research concentrating so far on the hard templating syntheses, in which the nanostructure of rigid biomaterials was replicated in the final Ht product [10,11,12,13,14,15,16,17,18,19]. In most works cellulosic polymers of various provenience have been used in the capacity of sacrificial biotemplates. In such a way formation of biomorphic hierarchical Mg-Al, Zn-Al and Ni-Al Ht was achieved, the materials proving to be excellent adsorbents of a variety of substances, including bovine serum albumin, oil, dyes and antibiotics [11,13,14,15,16,18]. Zhang et al. [12] demonstrated that advanced Zn-Al Ht/Al_2_O_3_ composites with tunable compositions and micro/nanostructures could be grown by structure replication of cellulose fibers. In some cases complex biological nanoarchitectures, such as those provided by pine pollen or surface of legume, were used, and resulted in hierarchical nanostructures with improved photocatalytic and/or catalytic performance [10,17,19].

In contrast, the use of sacrificial soft biotemplates, i.e., deformable systems based on organic molecules or supramolecules, remains, as yet, a virtually unexplored area, with only a couple of recent works addressing this issue [20,21], despite the known ability of Ht to form nanocomposites with soft biopolymers [22]. There is no doubt, however, that this methodology will soon gain on importance, since soft biotemplating procedures proved fruitful in the syntheses of nanostructured metal oxides [23,24,25,26].

The present work aims at getting deeper insight into the application of starch as a soft templating agent for the synthesis of nanostructured Ht materials. Nanocrystalline Ht solids can be obtained, for instance, from water-in-oil microemulsions, but the syntheses are very laborious and difficult to upscale [27,28,29,30]. The attractive feature of starch biopolymers is their “green” character, abundant availability, low cost, and facile gelation in aqueous medium [31]. Starch is a mixture of homopolymers of α-D-glucose: amylose (linear) and amylopectin (branched), and typically forms granules, which, depending on the biological origin, vary in shape, size, structure, and chemical composition. Properties of starch can be modified by physical methods, e.g., thermal treatment in various solvents, ultrasonication, microwave treatment, or chemically, by introducing new functional groups bringing about distinctive changes in the physicochemical properties of the polymer. All these features render starch templates particularly appealing for the purpose of materials design. This study concentrates on the effect brought about by the use of starch template, under various synthetic conditions, on the structural and compositional properties of prepared Mg-Al Ht materials. Alkali hydroxides in combination with appropriate alkali carbonates are the most frequently used precipitating agents for the synthesis of carbonate forms of Ht [1]. However, the resulting products usually contain alkali contaminants, which may be detrimental for some Ht applications. For instance, when used as solid base catalysts in biodiesel production, such materials may release alkali ions to the reaction mixture, thereby contaminating the product and diminishing the advantage of a heterogeneous versus homogeneous catalysis [32]. In such cases the use of an alkali-free synthetic procedure, e.g., by replacing alkali reagents with ammonia-based ones, is advised [33]. This aspect is a focus of the present study, because alkali-free synthesis enables direct thermal treatment of the as received dried Ht/starch mixtures.

## 2. Materials and Methods 

Synthesis of Ht with the intended Mg/Al ratio equal to 3 was carried out at room temperature, by co-precipitation at pH = 10, using 1 M aqueous solution of Mg and Al nitrates as the source of structure-forming cations, 7.5% NaOH or 10% NH_3_ aqueous solutions as precipitating agents, and Na_2_CO_3_ or (NH_4_)_2_CO_3_, as sources of carbonate anions, respectively. All chemicals were of p.a. purity, provided by POCH, Poland. The materials are further referred to as Ht^NaOH^ and Ht^NH3^. Syntheses in the presence of starch template were carried out by dissolving selected reagents in the gelatinized aqueous solution of starch prepared by heating 0.2 wt.% starch suspension in water at 95 °C for 3 h. Commercial potato (PPZ Trzemeszno, Trzemeszno, Poland), corn (Melvit SA, Warszawa, Poland), and cassava (Bio Planet SA, Leszno, Poland) starches, referred to as POS, COS, and CAS, were investigated. The synthesized materials are denoted Ht^precipitating agent^_wt. %starch type_, e.g., Ht^NH3^_0.2POS_ means Ht precipitated with ammonia-based reactants, in the presence of 0.2 wt.% POS gel. The precipitates were separated from the mother liquor by centrifugation, and dried in a drying box at 50 °C without washing. In order to check on the effect of method of drying, a selected as received sample was divided into two parts, of which one was dried in a drying box at 50 °C, the other, after solidification in liquid nitrogen, was freeze-dried in a CHRIST freeze dryer Alpha 1–4/LD. The effect of washing was studied by subjecting the as received freeze-dried Ht^NH3^_0.2POS_ precipitate to three washing/centrifugation cycles with distilled, not decarbonated water, of temperature 0, 25, 50 or 80 °C, followed by freeze drying of the resulting slurry.

X-ray diffraction (XRD) patterns were recorded using X’Pert PRO MPD (PANalytical, Almelo, The Netherlands) diffractometer with CuKα radiation (40 kV, 30 mA) selected by a nickel monochromator in the diffraction beam, with a step size 0.05°. Crystal sizes (the sizes of coherently scattering domains) of the Ht materials in the *c* and *a* direction of the unit cell, corresponding to the plate-like crystal thickness and lateral dimension, were estimated by analyzing the broadening of (003) and (110) reflections with Scherrer equation D_hkl_ = Kλ/βcosθ (D_hkl_—coherently scattering domain in the direction perpendicular to the (hkl) plane; K—shape factor, usually assumed 0.9, λ—incident ray wavelength; θ—Bragg diffraction angle and β—full width at half maximum of the (hkl) reflection, after correcting for the instrumental broadening), using the X’Pert High Score software (version 3.0, PANalytical, Almelo, The Netherlands). It must be noted that, in general, lattice disorder and/or strain, caused by, e.g., mechanical activation, if present, would also contribute to line broadening. Therefore the actual dimensions of coherently scattering domains may be somewhat larger than those obtained with the Scherrer estimate, but the latter serve well as indicators for comparative study of crystallinity trends. Whenever possible, the deconvolution of superimposed XRD reflexes was carried out with aid of the X’Pert High Score software. The uncertainty in calculation of the lattice parameters is < 0.05% of the determined value, the error of D_hkl_ determination about 10%.

Scanning electron microscopy–energy dispersive X-ray spectroscopy (SEM/EDS) analysis was carried out with aid of JEOL JSM-7500F microscope (JEOL, Tokyo, Japan) coupled with INCA PentaFetx3 EDS (Oxford Instruments, Abingdon, UK) system. SEM images were recorded for the uncoated samples deposited on 200 Mesh copper grids covered with carbon support film. 

Fourier transform infrared (FTIR) absorption spectra in middle infrared were recorded using transmission mode with Nicolet 6700 spectrometer (Thermo Scientific, Madison, WI, USA), in the 4000–400 cm^−1^ range. Samples were prepared as KBr pellets. 64 scans at 2 cm^−1^ resolution were taken for each sample. 

## 3. Results and Discussion

For reasons indicated in the Introduction, the research presented in this work concentrated on the use of NH_3_aq and (NH_4_)_2_CO_3_ as precipitating agents, but for the purpose of comparison, some Mg-Al Ht samples were also synthesized using the Na-based reagents. The data in Table 1 shows that the Ht materials obtained with NH_3_aq/(NH_4_)_2_CO_3_ reagents tended to display a somewhat lower Mg/Al ratio than those prepared with the use of NaOH/Na_2_CO_3_, which agrees with the previous report [34]. 

**Figure 1:**
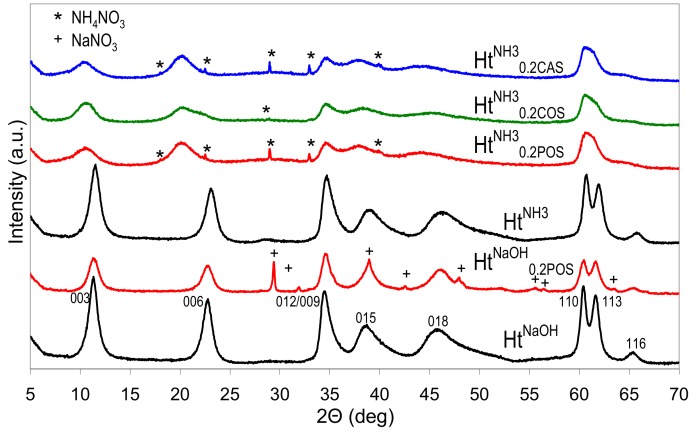
X-ray diffraction (XRD) patterns of Ht materials obtained with the use of different precipitating agents and in the presence or absence of various starches.

Figure 1 shows the XRD patterns of Ht^NaOH^ and Ht^NH3^ materials obtained by the standard co-precipitation, and of the appropriate Ht/starch composites prepared with the use 0.2 wt.% aqueous solution of POS gel instead of pure water, for preparation of all reagents. The diffractograms of Ht^NaOH^ and Ht^NH3^ samples are characteristic of a carbonate form of Ht-like structure, and the appropriate indexes of XRD reflections are given (ICSD ref. code 086655). The data on the d_003_ and d_110_ interplanar distances, and the crystal sizes along (003) (D_003_) and (110) (D_110_) directions are listed in Table 1. In the case of samples prepared in the presence of starch with NH_3_aq and (NH_4_)_2_CO_3_ the (110) and (113) reflections are broadened and overlap, making determination of d_110_ and D_110_ parameters impossible. It is apparent that the nature of the precipitating agent impacts Ht crystallinity, and the use of NaOH yields more crystalline materials, both in the absence and in the presence of starch.

In all cases syntheses carried out in the presence of starches result in materials with lower crystallinity than that of the appropriate reference prepared in the standard manner. In the adopted synthetic conditions, the effect is practically independent on the nature of used starch. Depending on the precipitant, the samples contain some NaNO_3_ or NH_4_NO_3_ impurity. Noteworthy, all Ht prepared in the presence of starches and precipitated with NH_3_aq are characterized by d_003_ values distinctly higher (0.836–0.847 nm) than those found in all other samples (0.770–0.785 nm). The basal spacing represents the sum of the layer and the interlayer thickness, and, therefore, is related to the size and orientation of charge balancing interlayer anions. The former values were closer to d_003_ characteristic of nitrate forms of Ht, while the latter point to the formation of Ht with carbonates in the interlayer [1]. Certain asymmetry of (00l) reflections visible in the XRD pattern of Ht synthesized in the presence of corn starch shows that the material was not quite homogeneous and part of the precipitate contains interlayer carbonate. Nevertheless, the predominance of the d_003_ around 0.84 nm indicates that, when NH_3_aq was employed as a precipitating agent, the presence of starch hindered the formation of carbonates despite the well known affinity of the hydrotalcite structures with Mg/Al ≤ 3 to intercalate carbonates as compensating anions [35]. It is known that, in the absence of intentionally added carbonate reagent, the use of ammonia precipitant, instead of NaOH, prevents intercalation of adventitious carbonate from atmospheric CO_2_ source and favors intercalation of nitrate [36]. In view of the much lower dissociation constant of ammonium carbonate vs. sodium carbonate, the effect has been attributed to the enhanced association of ammonium with carbonate, which limits availability of CO_3_^2−^ in solution, and prevents its intercalation [35]. However, the use of NH_3_aq in the presence of (NH_4_)_2_CO_3_ reagent does lead to the formation of predominantly carbonate form of Ht [37]. Findings of this study show, for the first time, that the preferential formation of nitrate-containing Ht may be achieved even in the presence of ammonium carbonate, provided a biopolymer is added to the solutions of ammonium reagents used for the synthesis. We tentatively attributed this phenomenon to the increased viscosity of the reaction medium, which favors topotactic intercalation of nitrate anions, introduced together with the layer forming cations, rather than reaction with carbonates, which have to diffuse to the site of Ht nucleation from further distance.

**Figure 2:**
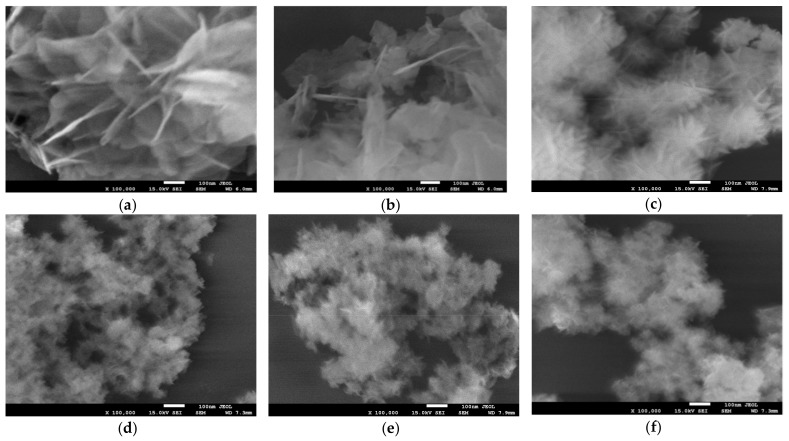
SEM images of Ht materials obtained with the use of different precipitating agents and in the presence or absence of various starches: (**a**) Ht^NaOH^; (**b**) Ht^NH3^; (**c**) Ht^NaOH^_0.2POS_; (**d**) Ht^NH3^_0.2POS_; (**e**) Ht^NH3^_0.2COS_ and (**f**) Ht^NH3^_0.2CAS_. Magnification × 100,000.

High resolution SEM analysis of Ht materials prepared in the standard manner and in the presence of starch reveals that the latter procedure has a significant impact on the Ht grain morphology. The effect is illustrated in Figure 2, which compares the images of materials, whose XRD patterns are shown in Figure 1. It is obvious that starch-containing environment exerts spatial restrictions on the formation of Ht grains, and results, in the case of ammonia-based synthesis, in particles of ca. 30–50 nm diameter, while Ht obtained in the absence of a biopolymer, consists of much larger platelets, with lateral dimension of several tenth micron order. The plate-like morphology is preserved in all synthesized materials. Notably, the observed effect of particle diminution parallels the trend observed for samples crystallinity, although particle size was always much larger than the size of the relevant coherently scattering domain. In particular, the use of a biopolymer template together with NH_3_aq/(NH_4_)_2_CO_3_ precipitating agent yielded finer nanoparticles than those produced with NaOH/Na_2_CO_3_. 

**Figure 3:**
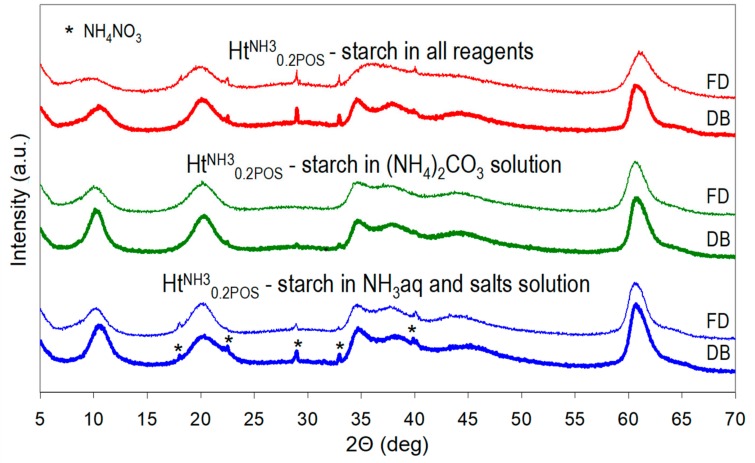
Impact of the method of starch addition (to all reagents, only to (NH_4_)_2_CO_3_, or to NH_3_aq + salt solutions) and of the method of drying (drying box—DB or freeze-drier—FD) on XRD patterns of HT^NH3^_0.2POS_.

A preparative procedure of Ht^NH3^_0.2POS_ sample was chosen for studying the effect of the manner of starch addition (all reagents vs. selected ones), of the method of drying (drying box vs. freeze drier) and of introducing the stage of washing of the as received material, on the properties of the final product. XRD patterns presented in Figure 3 and calculated crystal sizes in Table 2 show that to maximize the nanocrystalline character of Ht it was important that all reagents used for the synthesis were prepared in starch solution. The presence of starch in only selected reagents did reduce the crystal size with respect to that of the reference Ht^NH3^, but clearly to a lesser degree. Another important factor determining the structural features of synthesized Ht samples was the manner of drying. Freeze-drying was clearly the preferred method for achieving the possibly low Ht crystal size, because it helped to preserve the as-received crystallinity, while during conventional drying a degree of recrystallization occurred (Figure 3, Table 2). Moreover, the d_003_ basal spacings of freeze-dried samples were consistently slightly higher than those of their counterparts dried in a conventional manner. This indicates that during time spent by the wet cake in a drying box a degree of Ht carbonation occurs. 

**Figure 4:**
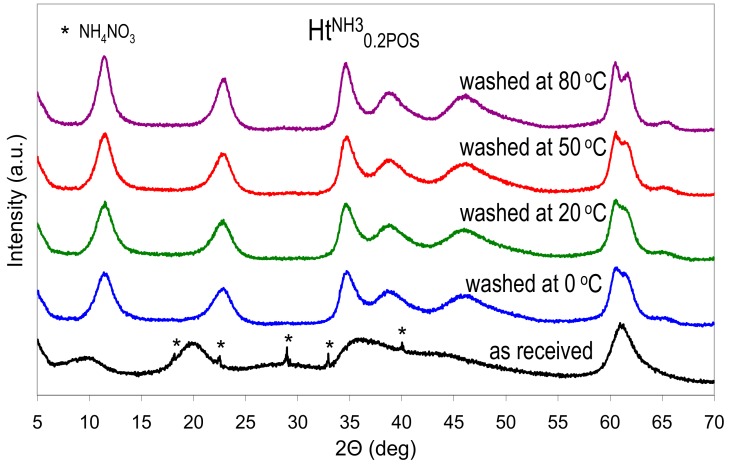
Structural changes in the Ht^NH3^_0.2POS_ sample upon washing with water at different temperatures. All materials freeze dried.

As stated earlier, if the Ht materials prepared in the presence of starch were precipitated with NH_3_aq, the washing of the as received samples in not necessary, because both starch and ammonium nitrate impurity will be removed during calcination. However, when the synthesis aims at preparation of uncalcined Ht samples, then a washing step has to be included in the preparative procedure, in order to obtain pure Ht phase. The starch polymer present in the as received precipitate is water soluble, hence it may be removed by washing with distilled water. The as received freeze-dried Ht^NH3^_0.2POS_ sample, with the lowest observed crystallinity, has been chosen to study the possible effect of washing. The sample has been subjected to washing with distilled water at various temperatures. The XRD patterns of the obtained materials are presented in Figure 4, while the calculated interplanar spacings, and crystal sizes are shown in Table 3.

A visible effect of washing, beside the disappearance of reflexes belonging to NH_4_NO_3_ impurity, was a shift of basal reflection from 0.897 nm characteristic of the as received sample, to 0.769–0.773 nm observed in washed materials, which points to the replacement of interlayer nitrates with carbonate anions. The effect was attributed to the presence of CO_2_ dissolved in the distilled water and extended contact of the slurries with air during washing/centrifugation cycles. Moreover, it was obvious that washing with water induced a rapid recrystallization of the as received Ht sample. A pronounced effect is visible already after washing carried out at 0 °C, and crystal size grows further with increase of the washing temperature. Thus, if washing with water is required, the use of ice-cold water is advised to minimize recrystallization.

It is of interest whether the effect brought about by starch consists only in providing a biopolymer framework preventing nanoparticles clustering, or there exists a stronger interaction between the template and the precipitated solid. To shed more light on this aspect of starch/Ht composite formation, the materials were subjected to FTIR analysis. 

**Figure 5:**
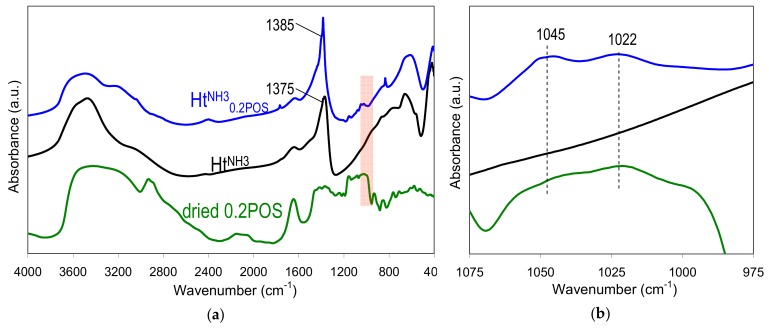
FTIR spectra of: (**a**) sample Ht^NH3^_0.2POS_, Ht^NH3^, and dried 0.2 wt.% potato starch gel, with the marked area indicative of starch order-disorder and the (**b**) blown-up fragment of the spectra in the marked area.

Figure 5 a shows the spectra in middle infrared range of Ht^NH3^_0.2POS_ material, Ht^NH3^ reference prepared without starch, and dried 0.2 wt.% gelatinized aqueous solution of starch used in the syntheses. The spectra of hydrotalcite phases are characteristic of these materials, with absorptions in the 3000–4000 cm^−1^ range due to the stretching vibrations of OH groups of the brucite-like sheets and in interlayer and/or adsorbed water, in the 1200–1800 cm^−1^ range due to interlayer anions and water, and below 1200 cm^−1^ due to lattice skeleton modes and low frequency bands of the interlayer anions [35]. The spectra confirmed the conclusion, drawn from the analysis of XRD patterns, that the Ht^NH3^_0.2POS_ sample contained predominantly nitrates in the interlayer, while Ht^NH3^ was intercalated chiefly by carbonates. FTIR spectrum of the former shows a 1385 cm^−1^ maximum characteristic of asymmetric stretching mode in nitrate anions, while the 1375 cm^−1^ absorption, dominating the spectrum of Ht^NH3^, was due to the asymmetric stretch in carbonates. The FTIR spectrum of dried starch gel shows bands due to OH stretches in water (3000–4000 cm^−1^), C–H stretching (2800–3000 cm^−1^), water scission and rocking vibrations (2000–2200 cm^−1^), water bending modes (1600–1700 cm^−1^), C–O, C–C, C–OH stretching (1100–1150 cm^−1^) and C–O–H bending (900–1100 cm^−1^) [38]. The latter region is referred to as a fingerprint one, because relative intensities of bands at 1045, 1022 and 1000 cm^−1^ have been shown to be sensitive to structural changes in starch. In particular, in amorphous starch the band at 1022 cm^−1^ tended to be more pronounced than the other two, while the 1045 and/or 1000 cm^−1^ gained on intensity in ordered starch samples. This section of FTIR spectra, marked in Figure 5a, was blown up in Figure 5b. It may be seen that dried potato starch gel shows a poorly resolved spectrum dominated by the maximum at 1022 cm^−1^, in accordance with the loss of structural order occurring upon high temperature gelation. On the other hand, in the Ht^NH3^_0.2POS_ sample a clear increase of the intensity of the 1045 cm^−1^ band in relation to the 1022 cm^−1^ one was observed. Such an effect may be taken as an indication that upon interaction with hydrotalcite nanoparticles a degree of short range molecular order is regained by the starch gel adhering to the surface of precipitated Ht. 

## 4. Conclusions

Results of the present study show that the synthesis of Ht materials in the presence of starch biotemplate, with use of the ammonia-based precipitant, enables preparation of nanocrystalline precipitates with very fine (< 50 nm) particle size, comparable with those obtained by means of the inverse micellar route, but at much lower cost and with significantly less effort. Under the adopted experimental conditions all investigated types of starch had a similar effect on the crystallinity and the grain size of the Ht precipitates. Freeze-drying was the preferred method for achieving the possibly low Ht crystal size. Ht with the smallest nanocrystals was obtained when starch was present in all solutions used during synthesis. Synthesis in the presence of starch gel hindered incorporation of carbonate anions, and the layer charge was preferentially compensated by nitrates from a metal salts solution. Washing with water induced rapid recrystallization of the as received Ht materials and caused an exchange of nitrates with carbonates. If the washing step is required, the use of ice-cold water was advised to minimize recrystallization. The FTIR spectra show that an interaction existed between the biopolymer template and the Ht particles, resulting in a higher degree of order within the starch component adhering to Ht surface. 

## Figures and Tables

**Table 1:** The effect of the precipitating agent and of the nature of starch on XRD determined d_003_ and d_110_ interplanar spacings, and D_003_ and D_110_ crystal sizes; EDS determined Mg/Al ratio.

Sample	Interplanar Spacing (nm)		Crystal Size (nm)		Mg/Al
	d_003_	d_110_	D_003_	D_110_	
Ht^NaOH^	0.780	0.1532	8.5	16.4	2.86
Ht^NaOH^_0.2POS_	0.785	0.1533	7.0	12.6	2.92
Ht^NH3^	0.770	0.1526	7.2	14.7	2.59
Ht^NH3^_0.2POS_	0.842	n.d.	3.3	n.d.	2.80
Ht^NH3^_0.2COS_	0.836	n.d.	4.1	n.d.	2.77
Ht^NH3^_0.2CAS_	0.847	n.d	3.6	n.d	2.68

**Table 2:** The effect of the method of starch addition and of the method of drying on XRD determined d003 interplanar spacings, and D_003_ crystal sizes.

Sample	d_003_ Interplanar Spacing (nm)		D_003_ Crystal Size (nm)	
	Drying Box	Freeze-drier	Drying Box	Freeze-drier
Ht^NH3^_0.2POS_ starch in all reagents	0.842	0.897	3.3	2.8
Ht^NH3^_0.2POS_ starch in (NH_4_)_2_CO_3_ solution	0.862	0.877	5.0	3.5
Ht^NH3^_0.2POS_ starch in NH_4_aq and in salts solution	0.834	0.872	4.1	3.8

**Table 3:** The effect of washing with water on XRD determined d_003_ and d_110_ interplanar spacings, and D_003_ and D_110_ crystal sizes of freeze-fried Ht^NH3^_0.2POS_ sample.

Sample	Interplanar Spacing (nm)		Crystal Size (nm)	
	d_003_	d_110_	D_003_	D_110_
Ht^NH3^_0.2POS_ as received	0.897	n.d.	2.8	n.d.
Ht^NH3^_0.2POS_ washed at 0 °C	0.770	0.1530	4.2	8.4
Ht^NH3^_0.2POS_ washed at 20 °C	0.769	0.1531	4.3	8.6
Ht^NH3^_0.2POS_ washed at 50 °C	0.770	0.1530	4.8	8.8
Ht^NH3^_0.2POS_washed at 80 °C	0.773	0.1531	5.6	10.2
